# Supplementary material for: Call for emergency action to limit global temperature increases, restore biodiversity, and protect health
Source: Lancet. 2021 Sep 11;398(10304):939–41. doi: 10.1016/S0140-6736(21)01915-2 (PMC8428481; doi:10.1016/S0140-6736(21)01915-2)
Supplement: Supplementary appendix [file mmc1.pdf]

THE LANCET

Supplementary appendix

This appendix formed part of the original submission. We post it as supplied by the authors.

Supplement to: Atwoli L, Baqui AH, Benfield T, et al. Call for emergency action to limit global temperature increases, restore biodiversity, and protect health. *Lancet* 2021; published online Sept 6. http://dx.doi.org/10.1016/S0140-6736(21)01915-2.

Appendix

Call for emergency action to limit global temperature increases, restore biodiversity, and protect health

The Comment Call for emergency action to limit global temperature increases, restore biodiversity, and protect health is being publish simultaneously in the following journals. This full list can also be found here, as well as a further list of supporting journals: https://www.bmj.com/content/full-list-authors-and-signatories-climate-emergency-editorial-september-2021

Acta Orthopaedica et Traumatologica Turcica
Advances in Nursing Science
Advances in Nutrition
African Journal of Laboratory Medicine
Afro-Egyptian Journal of Infectious and Endemic Diseases
Age and Ageing
Alcohol and Alcoholism
Allergy
Alpha Psychiatry
American Journal of Clinical Pathology
American Journal of Health-System Pharmacy
American Journal of Hypertension
American Society of Microbiology
Animal Bioscience
Annals of African Surgery
Annals of Behavioral Medicine
Annals of Oncology
Annals of Public Health
Annals of the Rheumatic Diseases
Annals of the Royal College of Surgeons of England
Archives of Disease in Childhood
Archives of the Turkish Society of Cardiology
Asia Pacific Journal of Public Health
Balkan Medical Journal
Belgian Journal of Medicine
Biosis: Biological Systems
BJOG
BMJ Case Reports
BMJ Evidence-Based Medicine
BMJ Global Health
BMJ Health & Care Informatics
BMJ Innovations
BMJ Leader
BMJ Military Health
BMJ Nutrition, Prevention & Health
BMJ Open
BMJ Open Gastroenterology

BMJ Open Ophthalmology
BMJ Open Quality
BMJ Open Respiratory Research
BMJ Open Science
BMJ Open Sport & Exercise Medicine
BMJ Paediatrics Open
BMJ Quality & Safety
BMJ Sexual & Reproductive Health
BMJ Supportive & Palliative Care
BMJ Surgery, Interventions, & Health Technologies
Bosnian Journal of Basic Medical Sciences
Brain
Brain Communications
British Dental Journal
British Journal of General Practice
British Journal of Ophthalmology
British Journal of Sports Medicine
British Medical Bulletin
Bulletin of the World Health Organization
Cadernos de Saúde Pública
Canadian Journal of Respiratory Therapy
Canadian Medical Association Journal
Cardiovascular Research
Caribbean Medical Journal
Chinese Science Bulletin
CIN: Computers, Informatics, Nursing
Clinical Medicine
Croatian Medical Journal
Crohn's & Colitis 360
Cureus Journal of Medical Science
Current Developments in Nutrition
Danish Medical Journal
Diseases of the Colon & Rectum
Dutch Journal of Medicine
East African Medical Journal
EBioMedicine
EClinicalMedicine
Emergency Medicine Journal
EP Europace
European Heart Journal
European Heart Journal - Acute Cardiovascular Care
European Heart Journal - Cardiovascular Imaging
European Heart Journal - Case Reports
European Heart Journal - Digital Health
European Heart Journal - Quality of Care and Clinical Outcomes
European Heart Journal – Cardiovascular Pharmacotherapy
European Journal of Cardio-Thoracic Surgery
European Journal of Cardiovascular Nursing
European Journal of Hospital Pharmacy
European Journal of Preventive Cardiology
European Journal of Public Health
Evidence-Based Mental Health
Evidence-Based Nursing
Family Medicine and Community Health
Family Practice
Finnish Medical Journal
Frontline Gastroenterology

Gaceta Sanitaria
Gastrointestinal Nursing
General Psychiatry
Global Health Action
Global Heart
Health Policy and Planning
Health Promotion International
Health Promotion Journal of Australia
Heart
Huisarts en wetenschap
Human Molecular Genetics
Human Reproduction
IJQHC Communications
Indian Journal of Medical Ethics
Indian Journal of Medical Research
Inflammatory Bowel Diseases
Injury Prevention
Innovation in Aging
Integrated Healthcare Journal
International Journal of Epidemiology
International Journal of Gynaecology & Obstetrics
International Journal of Gynecological Cancer
International Journal of Health Policy and Management
International Journal of Integrated Care
International Journal of Medical Students
International Journal of Nursing Studies
International Journal of Older People Nursing
International Journal of Pharmacy Practice
International Nursing Review
JAMIA Open
JMIR Public Health & Surveillance
JNCI Cancer Spectrum
Journal of Child Health Care
Journal of Clinical Pathology
Journal of Crohn's and Colitis
Journal of Epidemiology & Community Health
Journal of Health and Caring Sciences
Journal of Health, Population and Nutrition
Journal of Medical Ethics
Journal of Medical Genetics
Journal of Medical Imaging and Radiation Sciences
Journal of Nepal Paediatric Society
Journal of Neurology Neurosurgery & Psychiatry
Journal of Open Health Data
Journal of Pharmaceutical Health Services Research
Journal of Pharmacy and Pharmacology
Journal of Public Health
Journal of Surgical Case Reports
Journal of Surgical Protocols and Research Methodologies
Journal of the American Medical Informatics Association
Journal of the Medical Association of Thailand
Journal of the National Cancer Institute
Journal of the Norwegian Medical Association
Journal of the Royal Society of Medicine
Journal of Travel Medicine
Journal of Tropical Pediatrics
Journal of Turkish Society of Microbiology

Kafkas Universitesi Veteriner Fakültesi Dergisi
Khyber Medical University Journal
La Revista Universitas Medica
Lab Medicine
Medical Humanities
Medical Journal of Australia
Medical Mycology
Medwave
Nephrology Dialysis Transplantation
Neuro-Oncology Advances
Neuro-Oncology Practice
Neurology
New England Journal of Medicine
Nicotine & Tobacco Research
Nurse Author & Editor
Nursing Inquiry
Nutrition Reviews
Occupational and Environmental Medicine
Occupational Medicine
Oxford Open Climate Change
Oxford Open Immunology
Pacific Rim Journal of International Nursing Research
Paediatrics & Child Health
Palliative Medicine
Pan American Journal of Public Health
Pediatric Infectious Disease Society of the Philippines Journal
Pediatric Nursing
Pharmaceutical Journal
PLOS Medicine
Postgraduate Medical Journal
Psychiatry and Clinical Psychopharmacology
PTJ: Physical Therapy & Rehabilitation Journal
Revista de la Facultad de Medicina Humana
Revista de Saúde Pública
Rheumatology
RMD Open
Schizophrenia Bulletin
Schizophrenia Bulletin Open
Sexually Transmitted Infections
SLEEP
SLEEP Advances
Stroke and Vascular Neurology
The American Journal of Clinical Nutrition
The BMJ
The Gerontologist
The Journal of Climate Change and Health
The Journal of Nutrition
The Journals of Gerontology, Series A
The Lancet
The Lancet Child & Adolescent Health
The Lancet Global Health
The Lancet Microbe
The Lancet Planetary Health
The Lancet Psychiatry
The Lancet Public Health
The Lancet Regional Health - Americas
The Lancet Regional Health - Europe

The Lancet Regional Health - Western Pacific
The National Medical Journal of India
The Pan-American Journal of Ophthalmology
Thorax
Tobacco Control
Translational Behavioral Medicine
Tropical Journal of Pharmaceutical Research
Turkish Archives of Otorhinolaryngology
Turkish Archives of Pediatrics
Turkish Journal of Anaesthesiology and Reanimation
Turkish Journal of Biochemistry
Turkish Journal of Cardiovascular Nursing
Turkish Journal of Orthodontics
Turkish Thoracic Journal
Veterinary Record
VOICE
Western Journal of Emergency Medicine
Women's Healthcare: A Clinical Journal for NPs
World Journal of Pediatric Surgery
